# Ten Years of JFMK: Scientific Progress, Disciplinary Evolution, and the Future of Human Movement Research

**DOI:** 10.3390/jfmk11010028

**Published:** 2026-01-08

**Authors:** Giuseppe Musumeci

**Affiliations:** 1 Department of Biomedical and Biotechnological Sciences, Anatomy, Histology and Movement Sciences Section, School of Medicine, University of Catania, Via S. Sofia 87, 95123 Catania, Italy; g.musumeci@unict.it; 2 Research Center on Motor Activities (CRAM), University of Catania, 95123 Catania, Italy

## 1. Introduction

Ten years have already passed since we made this journal available to the academic community, I must say that after all the results we have achieved in these ten years, I was not wrong to launch and found this journal and for this I thank the Founder and Chairman of the MDPI Board, for believing in me and granting me this important mission and trust. Today our statistics are: 1465 Papers published; 288 Papers cited 10 times or more; 2.5 Current Impact Factor; 3.7 CiteScore; 40 H5-Index; CiteScore category rank Q2 in: Physical Therapy, Sports Therapy and Rehabilitation; Orthopedics and Sports Medicine; Anatomy; Rheumatology; Histology; Sport Sciences.

As the Journal of Functional Morphology and Kinesiology (JFMK) enters its tenth anniversary year, we are presented with an opportunity to reflect on a decade of scientific advances and editorial development. Since the publication of its inaugural issue in 2016, the journal has progressively consolidated its role as a multidisciplinary platform for research spanning functional anatomy, kinesiology, sports medicine, rehabilitation, and the broader spectrum of human movement sciences [1].

The foundational aims of JFMK have remained constant throughout its growth: to offer a comprehensive forum for the dissemination of experimental and theoretical studies, to promote full methodological transparency, and to encourage detailed reporting that enables reproducibility and cumulative scientific progress. Over the years, this mission has supported the emergence of a diverse yet coherent body of work, ranging from investigations into muscle structure, tissue mechanics, postural control, and neurodegeneration to research on strength development, athletic performance, adapted physical activity, and clinical rehabilitation. 

A decisive moment in this trajectory occurred in 2018, when JFMK underwent a substantial reorganization of its scientific sections. This restructuring not only mirrored the maturation of the field but also facilitated a clearer thematic identity for the journal. Further adjustments in 2023 refined this structure even more, merging closely related domains and strengthening the conceptual continuity between functional anatomy, musculoskeletal disorders, biomechanics, athletic performance, and exercise for health promotion. These editorial developments occurred in parallel with significant indexing milestones: inclusion in Scopus in 2018, PubMed and PubMed Central in 2021, and the Emerging Sources Citation Index in 2023. The journal’s first Impact Factor (2.6), received in 2024 [2], marked an important recognition of this sustained growth and the scientific contribution of its authors. Furthermore, our Editorial Board Members continue to expand and increase diversity. At present, the board consists of 88 international and multidisciplinary members, underscoring the strong global foundation that supports the journal’s growth, represents a wide range of regions, including Europe, North America, Asia, Oceania, and the Middle East.

The evolution of JFMK has unfolded against the backdrop of profound transformations in the study of human movement. During the past decade, kinesiology and functional morphology have progressively shifted from reductionist approaches toward integrative frameworks that view movement as the emergent result of interactions among anatomical structures, physiological mechanisms, neuromotor control, and environmental constraints. This conceptual shift has been accompanied by methodological advances that have expanded the boundaries of observational and experimental research. Tools such as wearable inertial sensors, portable force platforms, ultrasound imaging, electromyography, and infrared thermography have made it possible to investigate movement with a degree of ecological validity and temporal resolution that was previously unattainable. The availability of real-world data has, in turn, stimulated the development of new analytical strategies capable of capturing complexity, variability, and context-dependent phenomena.

Artificial intelligence has become one of the most transformative drivers in contemporary movement science [3]. Its applications range from automated extraction of anatomical features and quantitative gait analysis to the prediction of injury risk, rehabilitation trajectories, and athletic performance based on multimodal sensor data. Rather than replacing established biomechanical and physiological models, AI enhances our ability to detect subtle patterns, characterize non-linear behavior, and integrate information across scales and contexts. Its adoption, however, entails specific responsibilities. Challenges related to data quality, algorithmic bias, reproducibility, and interpretability must be confronted with the same rigor applied to experimental and clinical research. In this regard, *JFMK* remains committed to promoting responsible methodological standards and fostering the dialog between movement scientists, clinicians, engineers, and data scientists. The convergence between computational intelligence and domain expertise will likely shape the next decade of inquiry, opening new avenues for innovation while demanding careful scientific stewardship.

## 2. Looking Forward to 2026

As we look toward the future, several priorities emerge for JFMK. Strengthening methodological transparency and ethics remains central: reproducibility, advanced statistical approaches, and detailed reporting are essential for building cumulative knowledge. Interdisciplinarity will continue to be a defining feature, as meaningful scientific progress increasingly depends on integrating perspectives from anatomy, biomechanics, neurophysiology, rehabilitation sciences, sports medicine, and AI-driven analytics. The journal also intends to expand its support for early-career researchers, recognizing their role in shaping emerging research directions and sustaining the vitality of the field. It is also essential for the journal to support the editorial team in the dissemination of results and the promotion of special issues at international scientific conferences in the field of movement sciences. Finally, we aim to enhance the global reach of JFMK, promoting equitable scientific exchange and encouraging contributions from diverse research communities.

The tenth anniversary of the journal is ultimately a celebration of collective effort. The achievements of JFMK over the past decade are the result of the dedication of authors who have chosen this platform for their work, reviewers who have ensured the quality and rigor of published studies, Editorial Board Members who have guided the editorial process, and readers who have engaged critically with the scientific content, and the Managing Editor for his important help day after day. As Editor-in-Chief, I am deeply grateful for this shared commitment and honored to accompany the journal into its next decade.

Entering 2026, JFMK stands at the intersection of consolidated disciplinary knowledge, emerging theoretical paradigms, and technological innovation. The challenges and opportunities that lie ahead will undoubtedly stimulate new lines of inquiry and redefine the questions we ask about human movement, morphology, and function. 

We hope you will continue to share our enthusiasm for the journal as we work to further strengthen JFMK’s role within the scientific community. Your contributions, research articles, reviews, and Special Issue proposals, remain essential to the vitality and advancement of the journal.

I extend my sincere gratitude to our authors, readers, and reviewers, whose commitment ensures the quality and impact of our publications. I also wish to thank our editorial board members for their expertise, our managing editor and the entire editorial office for their continuous support, and the MDPI teams across our global offices for their invaluable contribution to the journal’s growth.

*The scientific world is changing rapidly, and we, as a journal and as a publisher, will always adapt to these changes in the service of the academic and scientific community.*

*Giuseppe Musumeci*
